# Nanobubbles Form at Active Hydrophobic Spots on the Luminal Aspect of Blood Vessels: Consequences for Decompression Illness in Diving and Possible Implications for Autoimmune Disease—An Overview

**DOI:** 10.3389/fphys.2017.00591

**Published:** 2017-08-15

**Authors:** Ran Arieli

**Affiliations:** ^1^Israel Naval Medical Institute, Israel Defence Force Haifa, Israel; ^2^Eliachar Research Laboratory, Western Galilee Medical Center Nahariya, Israel

**Keywords:** nucleation, stabilization, sheep, surfactant, endothel, decompression illness

## Abstract

Decompression illness (DCI) occurs following a reduction in ambient pressure. Decompression bubbles can expand and develop only from pre-existing gas micronuclei. The different hypotheses hitherto proposed regarding the nucleation and stabilization of gas micronuclei have never been validated. It is known that nanobubbles form spontaneously when a smooth hydrophobic surface is submerged in water containing dissolved gas. These nanobubbles may be the long sought-after gas micronuclei underlying decompression bubbles and DCI. We exposed hydrophobic and hydrophilic silicon wafers under water to hyperbaric pressure. After decompression, bubbles appeared on the hydrophobic but not the hydrophilic wafers. In a further series of experiments, we placed large ovine blood vessels in a cooled high pressure chamber at 1,000 kPa for about 20 h. Bubbles evolved at definite spots in all the types of blood vessels. These bubble-producing spots stained positive for lipids, and were henceforth termed “active hydrophobic spots” (AHS). The lung surfactant dipalmitoylphosphatidylcholine (DPPC), was found both in the plasma of the sheep and at the AHS. Bubbles detached from the blood vessel in pulsatile flow after reaching a mean diameter of ~1.0 mm. Bubble expansion was bi-phasic—a slow initiation phase which peaked 45 min after decompression, followed by fast diffusion-controlled growth. Many features of decompression from diving correlate with this finding of AHS on the blood vessels. (1) Variability between bubblers and non-bubblers. (2) An age-related effect and adaptation. (3) The increased risk of DCI on a second dive. (4) Symptoms of neurologic decompression sickness. (5) Preconditioning before a dive. (6) A bi-phasic mechanism of bubble expansion. (7) Increased bubble formation with depth. (8) Endothelial injury. (9) The presence of endothelial microparticles. Finally, constant contact between nanobubbles and plasma may result in distortion of proteins and their transformation into autoantigens.

## Introduction

Decompression illness (DCI) may occur during a prolonged deep dive, after exiting the water, or following any other reduction in ambient pressure, due to the formation of bubbles from supersaturated gas. The finding that in pure water, cavitation will only occur after a pressure reduction of about 200 atmospheres absolute (ATA) (Gerth and Hemmingsen, 1976; Hemmingsen, 1977) led to the concept that the expansion of decompression bubbles due to supersaturation of dissolved gas cannot take place without the presence of pre-existing gas micronuclei. Experimental support for the prevalence of gas micronuclei was forthcoming from studies in gelatin (Yount and Yeung, 1981) and in animals (Evans and Walder, 1969; Vann et al., 1980). However, tiny gas micronuclei are not stable, and according to the Young-Laplace equation they should dissolve out immediately. In the course of the past 70 years, a number of hypotheses have been proposed to explain the nature of these mysterious gas micronuclei. In the main, their stabilization was attributed either to hydrophobic crevices (Harvey et al., 1944) or to a coating of surface-active molecules (Fox and Herzfeld, 1954). An incompressible skin encompassing the gas micronuclei, or limited diffusion across this skin, was also purported to contribute to their stability. A twist to the notion of gas micronuclei stability was introduced by Goldman (2009, 2010), who added considerations of elasticity, stiffness, and free energy in the tissue surrounding the bubble. Goldman suggested that rather than achieving stability, micronuclei remain in existence for a relatively long period and are rejuvenated by renewed production. Stabilization, however, does not explain how these gas micronuclei were produced, the nucleation stage. The production of gas micronuclei was suggested to stem from tribonucleation, known also as viscous adhesion or frictional cavitation, cavitation which takes place on the separation of solid surfaces (Hayward, 1967). Opening of the blood vessels or the heart valves, as well as joint and tendon movements, might cause tribonucleation. It was suggested that these gas micronuclei may become trapped in the stabilization structure, remaining there to be activated after decompression. Following decompression, bubbles are observed mainly in the venous blood and rarely in the arterial circulation. It was commonly hypothesized that bubbles are formed not within the blood vessels but in the tissues, from where they enter the blood stream.

These notions regarding bubble nucleation and stabilization brought about the development of different decompression models. The variable permeability model (VPM) incorporated size distribution of bubbles formed after decompression of gelatin. In order to slow down diffusion to fit the rate of development of DCI, it was assumed that bubbles have a skin with varying gas permeability (Yount, 1979; Yount and Hoffman, 1986; Yount et al., 2000; Kuch et al., 2011). Wienke (1990, 2009), in his reduced gradient bubble model, assumed exponential size distribution of gas micronuclei enveloped in surfactants to enable them to achieve stability. Hydrophobic crevices were used by Tikuisis (1986) to model decompression bubbles, and hydrophobic crevices in blood vessels were suggested by Chappell and Payne (2006) as a model for the release of bubbles into the blood stream.

No supporting physiological data have ever been provided for any of the hypotheses regarding nucleation and stabilization of bubbles, or for their transfer from tissue to blood. So the question of the nucleation, stabilization and location of gas micronuclei continues to be shrouded in mystery.

## Nucleation and stabilization of bubbles on a hydrophobic surface

**Figure 1:**
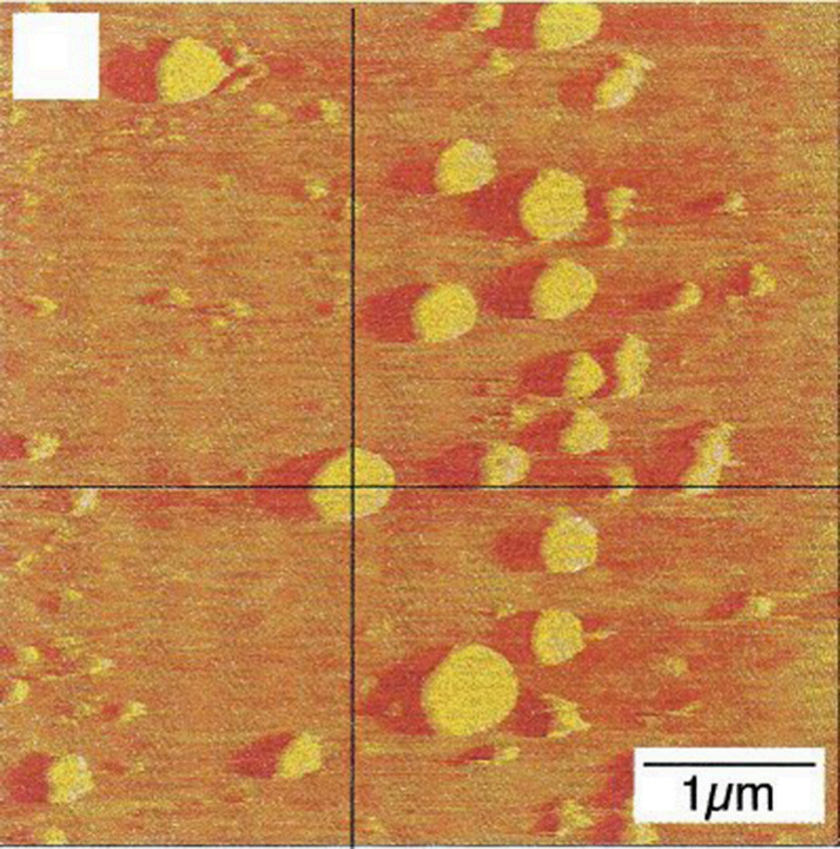
Evolution of nanobubbles on a hydrophobic surface. Nanobubbles which evolved from dissolved gas on a hydrophobic surface observed using atomic force microscopy (taken from Tyrrell and Attard, 2001 with permission).

Spontaneous formation of tiny flat gas nanobubbles 5–30 nm in diameter, on a smooth hydrophobic surface submerged in water containing dissolved gas, was first observed in the year 2,000 using atomic force microscopy (Ishida et al., 2000; Tyrrell and Attard, 2001; Switkes and Ruberti, 2004; Meyer et al., 2005; Stevens et al., 2005; Yang et al., 2007). An example of these nanobubbles taken from Ishida et al. (2000) is shown in Figure 1. Various theories have been proposed regarding their formation and stabilization. Brenner and Lohse (2008) suggested that bubbles are stabilized by a continuous influx of gas near the contact line, due to attraction of the gas to the hydrophobic walls. Seddon et al. (2011) suggested a Knudsen type, in which the generation of a bulk liquid flow effectively forces the diffusive gas to remain local. Weijs et al. (2012) and Weijs and Lohse (2013) proposed a molecular dynamics approach, with stabilization over many hours due to slowed diffusion and a gas layer at the solid surface. Bulavin et al. (2014) suggested that a vapor–liquid phase transition occurs at low temperatures, due to repulsive forces which increase the chemical potential of the molecules in the liquid phase near the hydrophobic surface. Fang et al. (2016) presented the time scale of nanobubble formation with intricate interplay among gas molecules, water, and hydrophobic solids. Whatever the mechanism underlying the nucleation and stabilization of nanobubbles, final doubts regarding an artifact of the atomic force microscopy were removed with optical confirmation (Karpitschka et al., 2012). In ultrasound irradiation, rectified diffusion increased the volume of the nanobubbles (Brotchie and Zhang, 2011), suggesting that they might expand in a state of gas supersaturation.

## Could these nanobubbles be the long sought-after gas micronuclei underlying decompression illness?

**Figure 2:**
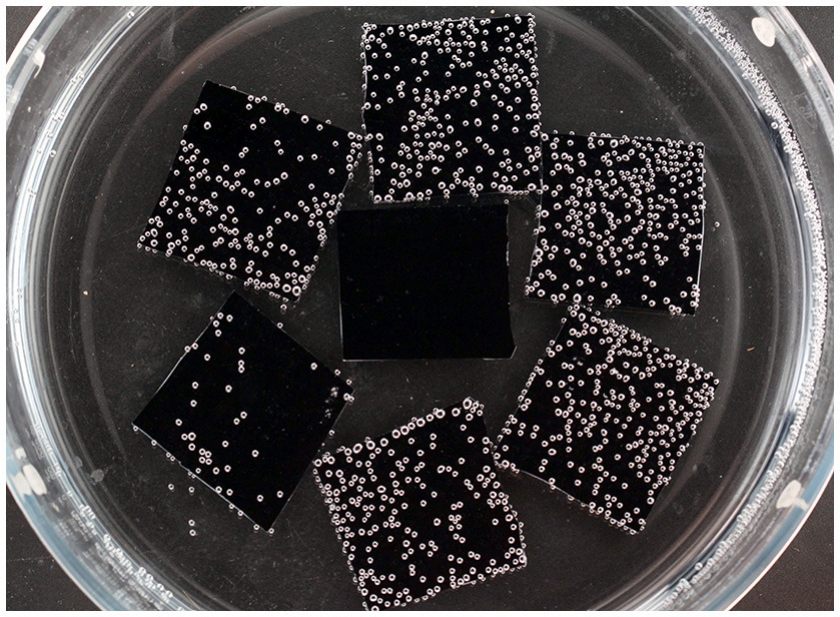
Expansion of decompression bubbles on a hydrophobic surface. Six hydrophobic wafers on the periphery and one hydrophilic wafer in the center, photographed 2.5 h after decompression from 300 kPa (20 m sea water) (taken from Arieli and Marmur, 2013a with permission).

We hypothesized that the nanobubbles which evolve on a hydrophobic surface may be the long sought-after gas micronuclei underlying decompression illness. Silicon wafers were coated with a single molecular hydrophobic layer. Hydrophobic and hydrophilic (non-coated) wafers were exposed overnight to hyperbaric pressure under double distilled water (after degassing). After decompression, the bowl containing the wafers was removed from the hyperbaric chamber for photography. Large numbers of bubbles appeared on the hydrophobic, but none on the hydrophilic wafers (Arieli and Marmur, 2011, 2013a). An example is shown in Figure 2, where the central wafer is hydrophilic and those on the periphery are hydrophobic. Bubbles detached from the wafer at a mean diameter of 4.2 mm, which agrees with the calculated attraction force for hydrophobicity with a contact angle of 90 degrees.

## Active hydrophobic spots (AHS) on the luminal aspect of blood vessels

**Figure 3:**
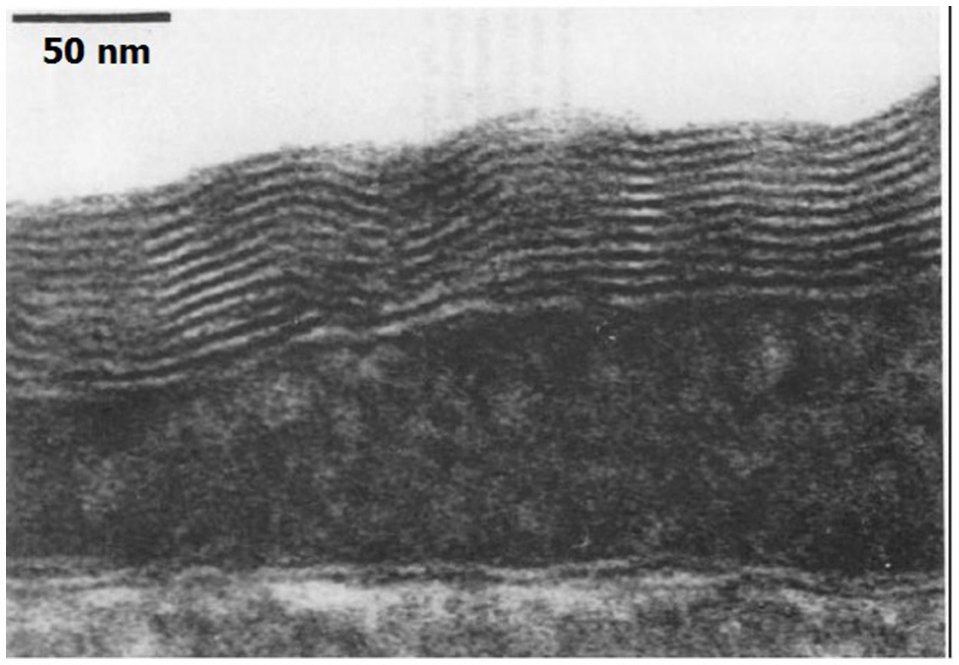
Phospholipids on the capillary. Electron microscope of the luminal aspect of a cerebral capillary from a sheep. An oligolamellar lining of phospholipids can be seen (taken from Hills, 1992 with permission).

Hills and Butler (1981) found that surfactants were present in the pulmonary circulation of dogs. Hills (1992) later demonstrated an oligolamellar lining of phospholipids on the luminal aspect of blood vessels in sheep. This lining was observed in various blood vessels, including the cerebral capillaries. He also noted hydrophobicity on the luminal aspect of blood vessels, and suggested that the source of the phospholipids was in lung surfactants. An example of the oligolamellar lining in a cerebral capillary is shown in Figure 3.

The combination of the early findings of Hills and our experiments on silicon wafers provided the impetus for our study of ovine blood vessels (Arieli and Marmur, 2013b, 2014, 2016; Arieli et al., 2015, 2016). The findings from these investigations eventually led us to propose a new physiological model for designing decompression procedures (Arieli and Marmur, 2017).

**Figure 4:**
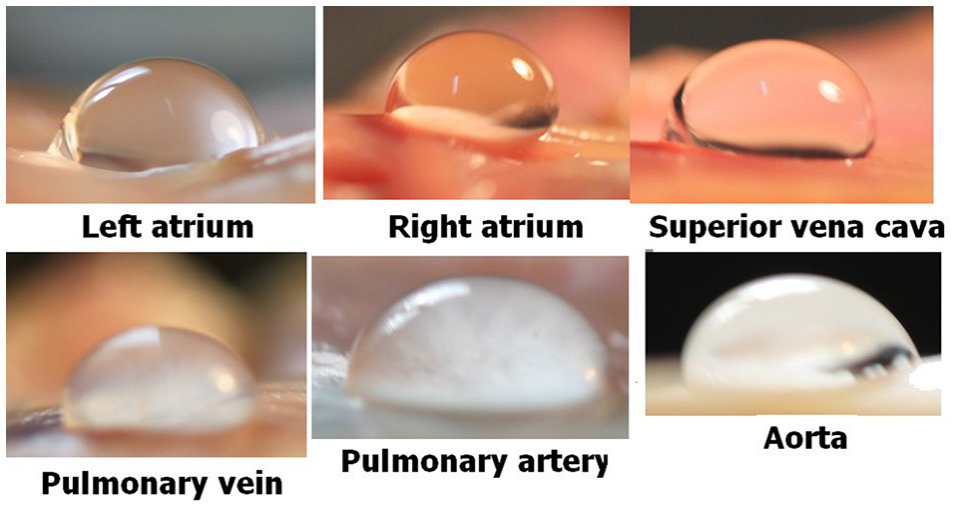
Hydrophobicity on blood vessels. Hydrophobicity was demonstrated at specific locations using a small drop of saline and a large contact angle (taken from Arieli and Marmur, 2013b with permission).

**Figure 5:**
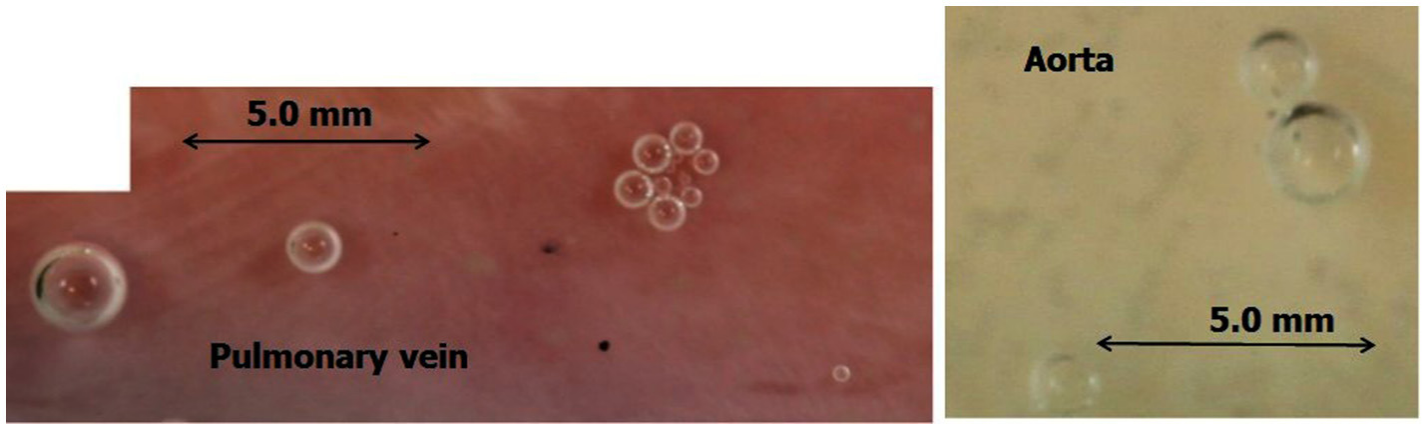
Bubble-producing spots. An example of defined spots, as revealed by bubble development from a pulmonary vein and aorta. Some spots produced just one bubble at a time, whereas others produced several bubbles at once.

**Figure 6:**
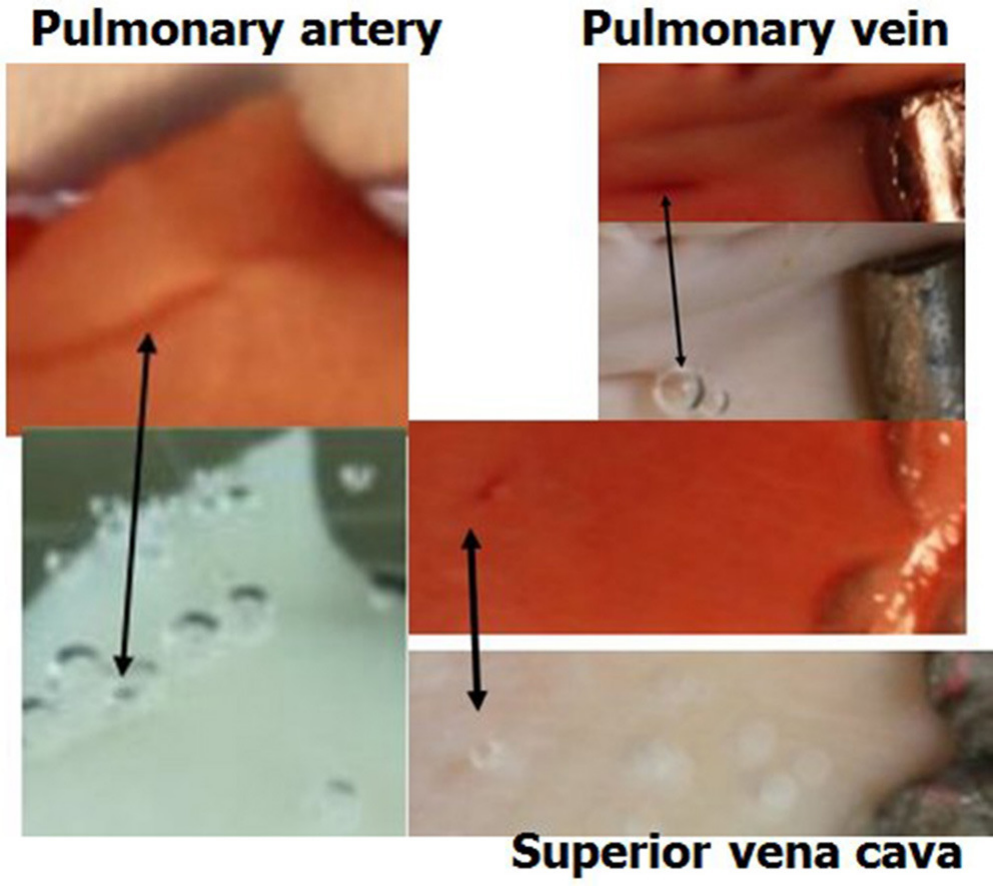
Active spots are hydrophobic. Samples of blood vessels photographed under saline, revealing the AHS at which bubbles nucleate and expand. The same sample after staining for lipids (dark red) is presented above its previous presentation. Arrows indicate the bubbles on the AHS and the corresponding red staining for lipids.

The general experimental set-up for the sheep blood vessel studies will be presented briefly here. We demonstrated hydrophobicity using a small drop of saline, checking the contact angle at specific locations on all the types of blood vessels examined. An angle above 100 degrees is considered hydrophobic (Figure 4). Various large blood vessels, the aorta, superior vena cava, pulmonary vein, and pulmonary artery, were obtained anaerobically from slaughtered sheep. Samples of the blood vessels were gently stretched on microscope slides under saline at the bottom of a Pyrex bowl, with the luminal aspect exposed. The bowl was placed in a cooled high pressure chamber at 1,000 kPa for about 20 h. Following decompression, the bowl was removed from the chamber for photography. Bubbles evolved and became visible at definite spots in all the six types of blood vessels examined, with large variability between sheep and between blood vessels. There was no visual indication as to the presence of an active spot until bubbles appeared. An example of these spots may be seen in Figure 5. Some spots produced one bubble at a time, whereas others produced several bubbles at once. On completion of the photographic session, the blood vessels were stained for lipids using Oil Red O. The bubble-producing spots stained positive for lipids (Figure 6). Henceforth, these spots were termed “active hydrophobic spots” (AHS).

**Figure 7:**
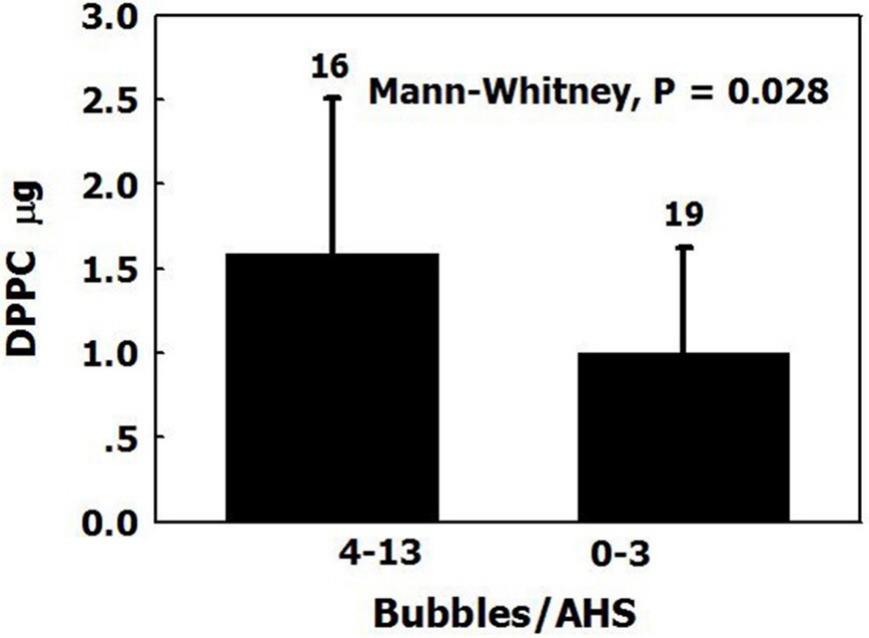
DPPC on blood vessels. The amount of DPPC (mean ± *SD*) in AHS which produced more than four bubbles over 30 min, compared with those which produced <4 bubbles and control samples (adapted from Arieli et al., 2016 with permission).

Dipalmitoylphosphatidylcholine (DPPC) is the main surfactant in the lung, representing 40% of total pulmonary surfactants. DPPC was found in the plasma of sheep (2.04 ± 0.90 μg/ml), and there was more DPPC in AHS which produced 4 or more bubbles within half an hour than in AHS which produced <4 bubbles (Figure 7). DPPC has a higher compaction capacity than other phospholipids, because its apolar tail is less bent (Hills, 1999). Surfactants tend to settle close to each other from a dilute solution to create aggregates (Sharma et al., 1996). We suggested that the surfactant DPPC leaks from the lung into the plasma and is transported through the circulation by albumins, finally settling on the luminal aspect of arterial and venous blood vessels and capillaries. There it forms an AHS, which is composed of an oligolamellar lining of DPPC and perhaps other constituents. Nanobubbles nucleate and remain stable on the AHS.

**Figure 8:**
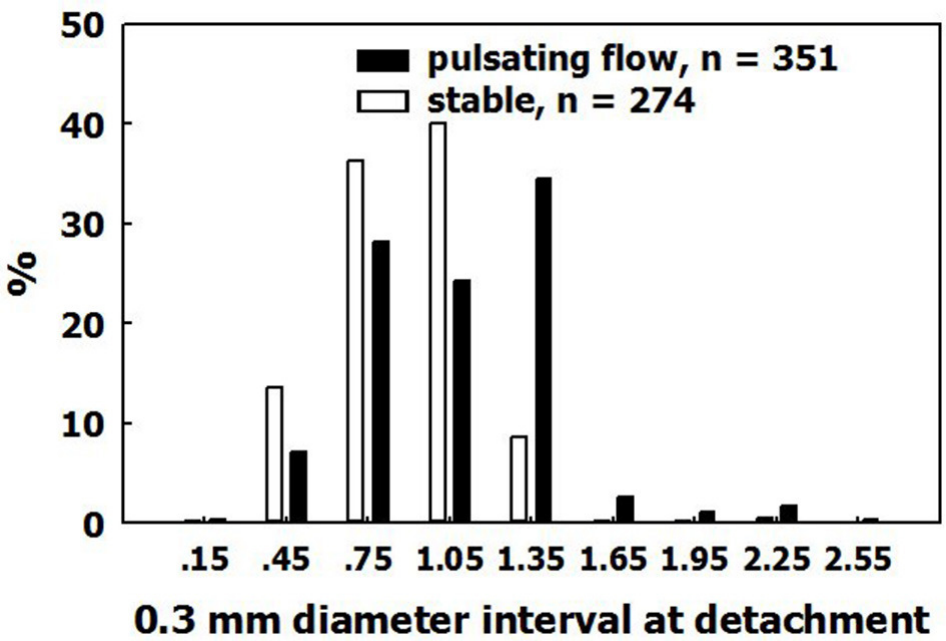
Size on detachment. The distribution of bubble diameters on detachment, in pulsatile flow and in stable conditions.

**Figure 9:**
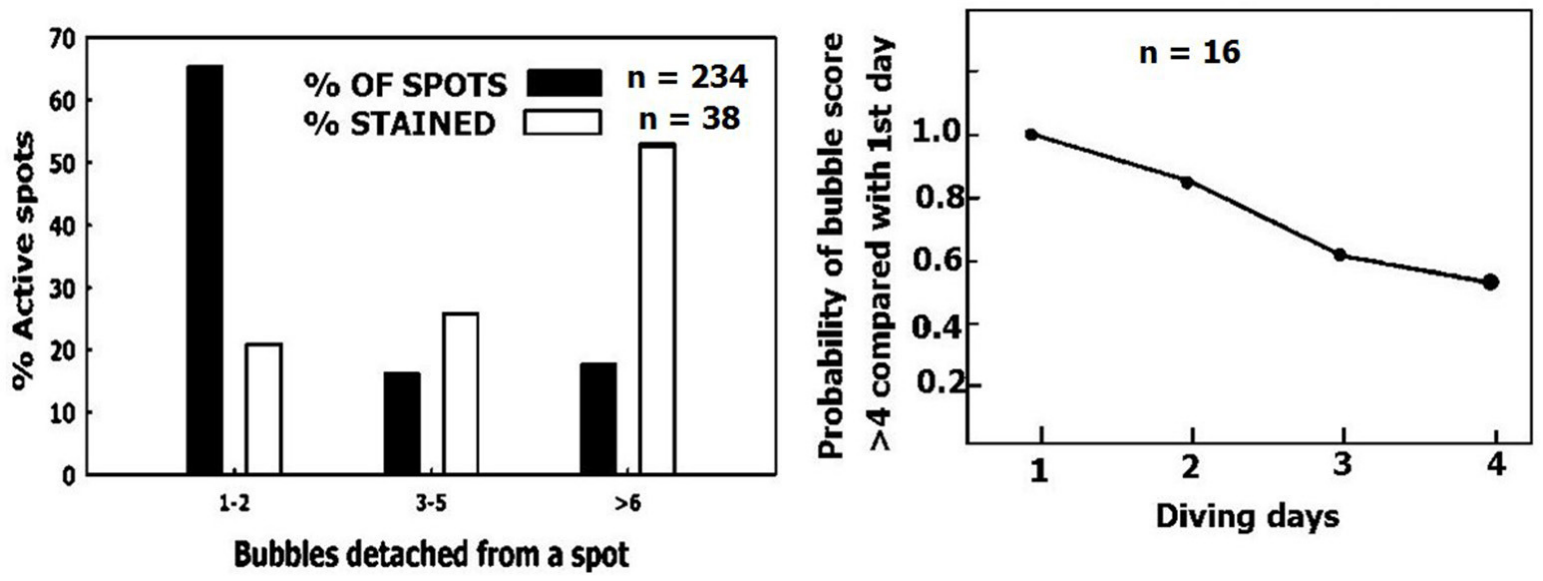
Removal of small sized AHS. **(Left)** Distribution of AHS (black columns) and the distribution of AHS staining positive for lipids (empty columns), as a function of their bubble production. **(Right)** The odds ratio value of having a higher bubble score over 4 consecutive days of diving compared with day 1 (adapted from Zanchi et al., 2014 with permission).

The fate of the expanding bubbles following decompression was studied under stable conditions, and in pulsatile flow with a mean velocity of 234 cm/min. This is greater than the flow velocity in the majority of blood vessels except for the very large ones. Bubbles continued to expand until detachment. Diameter on detachment is shown in Figure 8. Bubbles detached at a mean diameter of 0.99 ± 0.36 mm in pulsatile flow, and at a diameter of 0.81 ± 0.34 mm in stable conditions. Very few bubbles detached at a diameter below 0.6 mm, and almost none detached at a diameter smaller than 0.4 mm. Bubbles detached from the AHS at a diameter that was ~1.0 mm smaller than the detachment diameter from a hydrophobic silicon wafer (~4.2 mm). The buoyancy force calculated from the volume of the bubble on detachment from a hydrophobic silicon wafer agrees with the calculated adhesion force for hydrophobicity with a 90° contact angle. There are two possible explanations for the detachment of bubbles from AHS on the blood vessels at a smaller volume compared with hydrophobic silicon wafers. Either the AHS are small and irregular, having a perimeter that enables only limited contact between bubble and tissue, or it may be that the underlying phospholipids become detached along with the bubble. Support for the second assumption may be found in a number of phenomena related to the AHS, bubbles, decompression, and diving. (1) Bubbles that detach from large AHS, which produce several bubbles at once, are no larger than bubbles that detach from AHS which produce only one bubble at a time. (2) Subsequent staining for lipids failed to show most of the AHS which produced only one or two bubbles (Figure 9, left panel). The explanation for this was that phospholipids were carried away from the AHS along with the bubbles, leaving a smaller AHS which was no longer active. (3) After diving, microparticles may be observed in the blood, some of them composed of stripped endothelial membranes (Thom et al., 2011). (4) Some of these microparticles are enlarged, containing gas (Yang et al., 2012). I would suggest that detached bubbles carry with them pieces of membrane. Some lose their gas in the lung and are transformed into microparticles, whereas others retain some of their gas and remain as the enlarged microparticles. (5) Diving caused a reduction in endothelial function (Obad et al., 2007; Madden et al., 2010), indicating a damaged endothelium. (6) Endothelial damage (functional and anatomical) due to decompression bubbles was demonstrated in the pulmonary artery of the pig (Nossum et al., 1999) and the rat (Zhang et al., 2016). (7) The point of contact between the membraneous bilayer is prone to cavitate in clinically used ultrasound (Krasovitski et al., 2011), which suggests a weak adhesion force.

**Figure 10:**
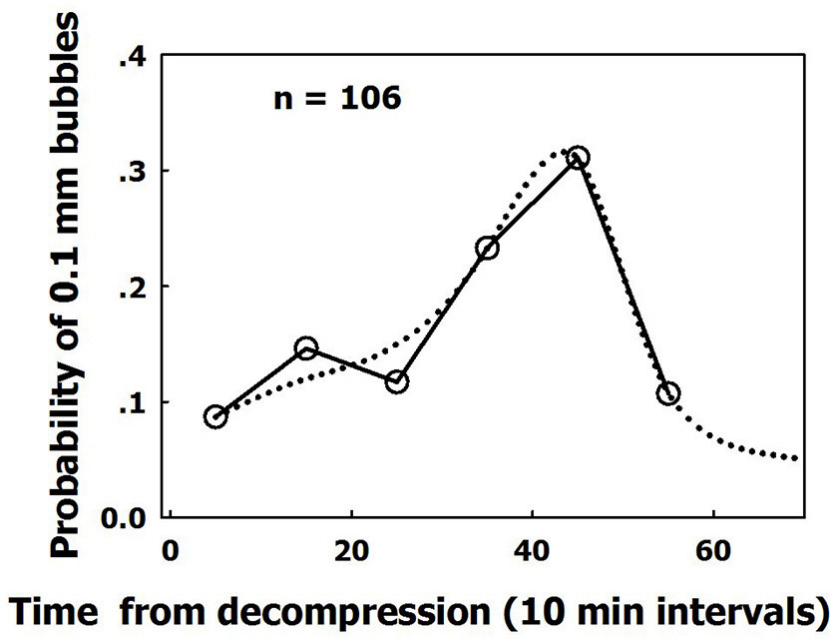
Dispersed initiation of AHS. Frequency of initiation of active hydrophobic spots (AHS). Initiation is defined as the moment the first bubble from an AHS reaches a diameter of 0.1 mm, as a function of time from decompression. The dotted line is a suggested smooth function (taken from Arieli and Marmur, 2017 with permission).

The smallest observable bubble in our studies (seen after magnification as a bright spot) was 50 μm in diameter. It took anything from just a few minutes up to about 20 min for a bubble to expand from a diameter of 0.1 mm to its size on detachment. The pattern of this second phase of growth agreed with simple diffusion. However, there was variability in the time from decompression until the first bubble evolved on an AHS and reached a diameter of 0.1 mm. The appearance on an AHS of the first bubble having a diameter of 0.1 mm peaked 45 min after decompression (Figure 10). This first slow phase, during which nanobubbles developed into gas micronuclei and started expanding, was therefore termed the “initiation phase.”

## Compatibility of AHS with decompression from diving

### Individual sensitivity to DCI

**Figure 11:**
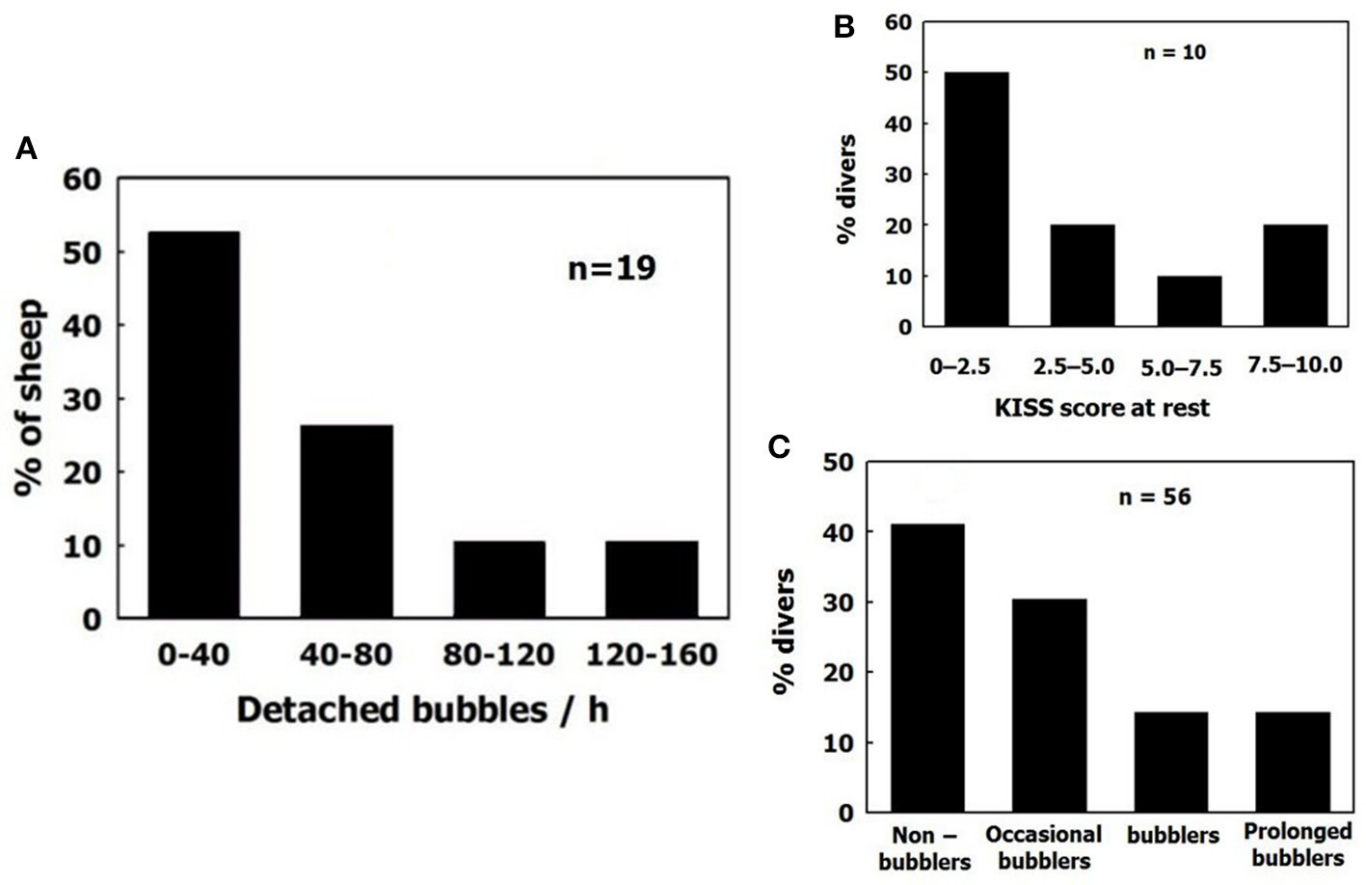
Distribution of bubblers—non bubblers. **(A)** In blood vessels of sheep. **(B)** (data compiled from Lambrechts et al., 2013) and **(C)** (data compiled from Cialoni et al., 2015 with permission), in divers.

Variability between divers (bubblers compared with non-bubblers) is similar to the variability between sheep, as depicted in Figure 11. Sheep were grouped according to total bubble formation for all the blood vessels examined in each animal, normalized for area and time. Divers are presented according to their bubble score (data compiled from Lambrechts et al., 2013; Cialoni et al., 2015). The distribution is similar, with a high frequency of non-bubblers and a low frequency of heavy bubblers.

### Age and acclimation

It has been suggested that the AHS are composed of lung surfactants deposited on the luminal aspect of blood vessels (Hills, 1992; Arieli, 2015). We recently confirmed the validity of this hypothesis, by showing that DPPC leaks from the lungs into the blood and settles to form AHS on the vessel (Arieli et al., 2015).

#### Age

Advancing age is a known risk factor for DCI and decompression bubbles (Carturan et al., 2002; Boussuges et al., 2009; Blatteau et al., 2011). It was shown that this increased sensitivity is age- and not fat-related (Schellart et al., 2012). We suggest that the number and area of AHS increase with age as a result of additional deposits of surfactants throughout life. This may explain the elevated risk of DCI and decompression bubbles with age.

#### Acclimation

Experienced divers will produce fewer bubbles than novice divers, and will thus be less prone to decompression sickness (Sayer et al., 2008; Pontier et al., 2009; Zanchi et al., 2014). Although, there were more AHS which produced just a few bubbles than AHS which produced large numbers of bubbles (Figure 9, left panel, black bars), the distribution of AHS which stained positive for lipids was diametrically opposed to this (Figure 9, left panel, empty bars). Our interpretation is that the substrate was carried away by detached bubbles (Arieli et al., 2016), inactivating the small AHS. An example of acclimation to diving is presented in Figure 9, right panel (adapted from Zanchi et al., 2014). The bubble score is reduced in subsequent dives. Acclimation to diving, as seen in experienced divers who run less risk of DCI and produce low grade bubbles, may be related to the depletion of phospholipids from the AHS by bubbles in previous dives.

### Increased risk of DCS in the second dive on the same day

**Figure 12:**
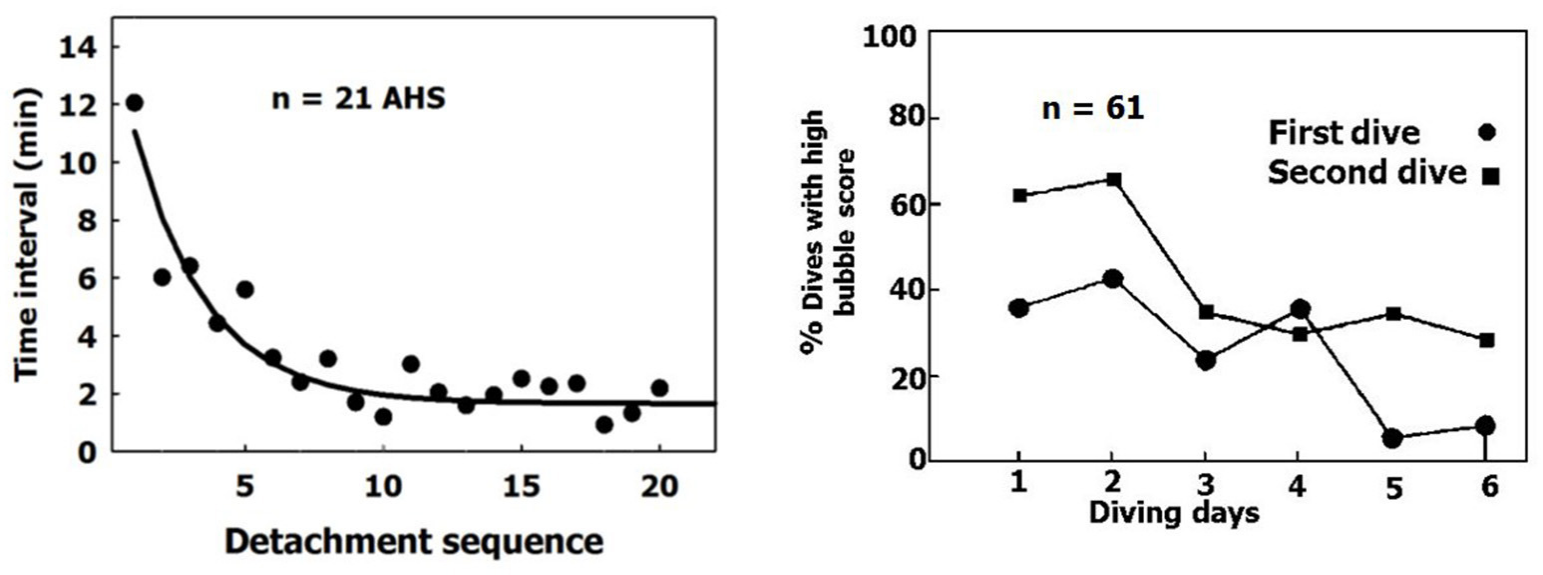
Activation of AHS. **(Left)** Time intervals between bubble detachments from the AHS as a function of the sequence of detachment from the same AHS. The first value is the time from decompression (adapted from Arieli and Marmur, 2014 with permission). **(Right)** Percentage of high bubble grades after the first and second dives on the same day (adapted from Dunford et al., 2002 with permission).

When a sequence of bubbles becomes detached from an AHS, the AHS is activated and the rate of bubble production increases (Figure 12, left panel). After seven successive detachments, the time interval between further detachments stabilizes over a short period. This activation of the AHS coincides with the phenomenon whereby more bubbles are seen in the blood after a second dive on the same day made using the same profile (Figure 12, right panel, adapted from Dunford et al., 2002). There are more activated AHS in the second dive.

### Arterial bubbles, neurological DCI, and spinal DCI

Because AHS are to be found in blood vessels within the arterial as well as the venous circulation, with a distribution going as far as the cerebral capillaries (Hills, 1992), bubbles might develop within the arteries. This mechanism can explain symptoms of neurologic decompression sickness that occur without arterialization of venous blood, either via a patent foramen ovale or intrapulmonary arteriovenous anastomoses (Madden et al., 2015; Balestra and Germonpre, 2016). After decompression, at any distal bifurcation of the arterial tree, flow decreases for each vessel and the vessel's diameter is reduced. This increases the surface area available for diffusion with respect to blood volume, and the reduced wall thickness reduces the diffusion barrier. Thus, the diffusion of inert gas from the tissue into the blood will rise along the arterial tree. This may cause the expansion of bubbles at AHS within the distal arteries. Local reduction of blood flow would enhance the diffusion of inert gas into the artery. If arterialization of venous blood were the source of arterial bubbles, because the brain receives eight times the amount of arterial blood directed to the spinal cord, more arterial bubbles shunted from the venous circulation should reach the brain (Hallenbeck et al., 1975). However, spinal DCI is over three times more frequent than cerebral DCI. The internal veins which drain the spinal cord do not have valves (Stringer et al., 2012). It was also suggested that after decompression, spinal blood flow is reduced due to obstructions in the epidural vertebral veins (Hallenbeck et al., 1975). It is therefore possible that decompression bubbles develop in distal arteries within the nervous system, and more so in the spinal cord. A detailed discussion of arterial bubbles was presented in the paper by Arieli and Marmur (2017).

### Preconditioning before diving to reduce decompression bubbles

Various methods have been established as pretreatment before diving to reduce decompression stress. It has previously been hypothesized that exposure to hyperbaric oxygen at the beginning of a dive would result in replacement of the inert gas in the micronuclei by oxygen, with subsequent consumption of the oxygen by the mitochondria. This would shrink gas micronuclei having the potential to grow into bubbles and thus reduce the risk of DCI. The preoxygenation hypothesis was supported by experimental studies in prawns, rats and humans (Arieli et al., 2009; Castagna et al., 2009, among others). Pre-dive exercise, sauna, and vibration all resulted in a reduced bubble score following the dive (Blatteau et al., 2007, 2008; Germonpré et al., 2009; Balestra et al., 2016; Germonpre and Balestra, 2017). One of the commonly suggested explanations for the relief obtained using these methods is elimination or dislodgment of some of the gas micronuclei from the surface of blood vessels. According to Fang et al. (2016), it takes somewhat more than 2 h for nanobubbles to reappear. Thus, pretreatment was carried out within the time scale required to preempt the renewal of nanobubbles, and consequently the development of gas micronuclei.

### Bubble growth rate

Previous models of decompression presented investigators with difficulty when it came to matching the time required for bubble expansion by diffusion (a short process) with the development of DCI (a prolonged process). To overcome this problem, some investigators suggested an artificially low diffusion constant (Hugon, 2014), while others suggested a bubble skin as a barrier to diffusion (Yount, 1979; Yount and Hoffman, 1986; Yount et al., 2000; Kuch et al., 2011) or an envelope of surfactants (Wienke, 1990, 2009). A bi-phasic mechanism of bubble expansion, initiation (Figure 10) followed by diffusion-driven growth, against the background of the AHS, makes bubble expansion compatible with the development of DCI.

### Bubble score increases with exposure pressure

**Figure 13:**
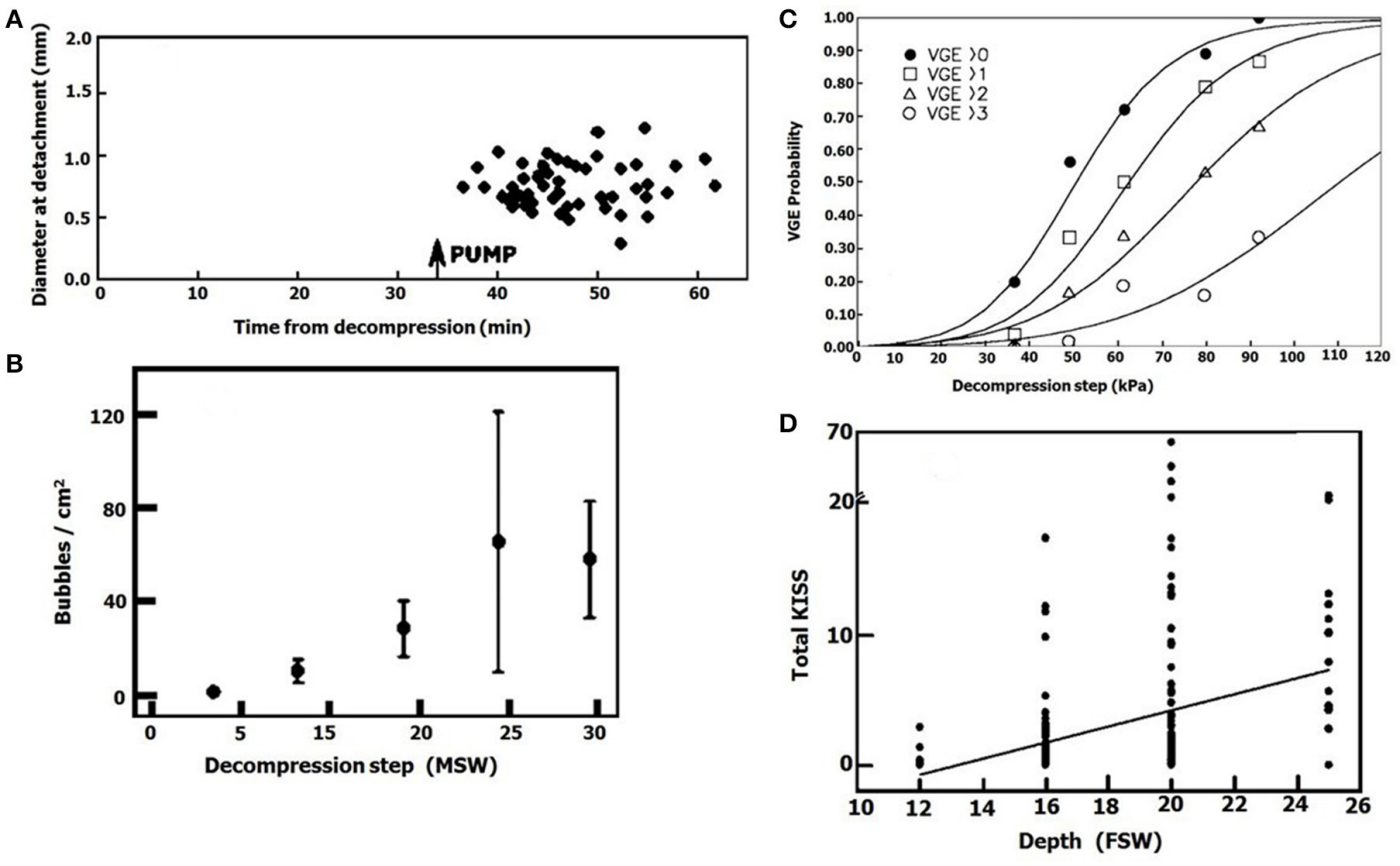
Effect of exposure pressure on bubble formation. **(A)** Diameter of bubbles which detached from an ovine blood vessel plotted against time from decompression. The first period before pump operation (gas tension in the proximity of the vessel 228 kPa), and the second after pump operation (gas tension in the proximity of the vessel 600 kPa). **(B)** Density of decompression bubbles on a hydrophobic silicon wafer as a function of exposure pressure (taken from Arieli and Marmur, 2013a with permission). **(C)** Bubble score in a diver after decompression from various pressures (adapted from Eckenhoff et al., 1990 with permission). **(D)** Bubble score after decompression from various depths (adapted from Cameron et al., 2007 with permission).

It is well-known that an increase in the exposure pressure in diving causes an increase in bubble production after decompression. An example of this is presented in Figures 13C,D (adapted from Eckenhoff et al., 1990; Cameron et al., 2007). It may be related to (1) Faster expansion of bubbles when there is a greater difference in gas tensions, with earlier detachment and thus an increase in the rate of bubble formation at the same AHS. (2) Activation of more AHS when gas tensions are higher. When a decompressed ovine blood vessel was taken from the hyperbaric chamber and placed in calm conditions, with a calculated gas tension of 228 kPa in the proximity of the tissue, no bubbles appeared. After activation of pulsatile flow of the saline, mixing yielded a gas tension of 600 kPa in the proximity of the tissue, and large numbers of bubbles appeared (Figure 13A). This was seen in 40% of the blood vessels, and we interpreted it as activation of gas micronuclei at the high gas tension. This is supported by our finding in the hydrophobic silicon wafer, that the density of bubbles increased with the decompression steps (Figure 13B). There will therefore be increased activation of AHS with the higher gas tensions when diving at elevated pressure.

### Endothelial injury

Contact between bubbles and signaling receptors at the endothelial membrane can promote unwanted events. Sobolewski et al. (2011, 2012) exposed endothelial cells to contact with air bubbles. They demonstrated an influx of calcium through a stretch-activated channel, such as a transient receptor potential vanilloid family member, triggering the release of calcium from intracellular stores. They also showed activation of a calcium-independent, PKCα-dependent signaling pathway, resulting in mitochondrial depolarization. Mitochondrial dysfunction is likely a key contributor to the pathophysiology of DCI. Bubble contact with endothelial cells released microparticles which decreased cell viability, increased apoptosis, and caused over-expression of pro-inflammatory cytokines (Yu et al., 2017). Protection of the endothelium with escin (the main active compound in horse chestnut seed extract) reduced the incidence of DCI in the rat (Zhang et al., 2017a). Bubbles which develop at the AHS on the surface of the endothelium are liable to come into contact with the cell membrane. In our pulsatile flow experiments, large bubbles which rocked back and forth with the flow most likely did come into contact with the endothelial membrane. Proteoglyans, glypicans, and glycoproteins (the glycocalyx) protrude some tens of nm from the luminal aspect of the membrane. Plasma proteins bind to the glycocalyx to produce a 0.5–1.0 μm layer of macromolecules. This endothelial surface layer is removed by reactive oxygen species (Pries et al., 2000). It is possible that reactive oxygen species in diving remove this layer, exposing more AHS to the flow of plasma and thus enhancing bubble production. Markers of endothelial injury, such as cell adhesion molecule-1 (ICAM-1) and endothelin-1 (ET-1), were well correlated with the timing of bubble detachment and their number in the decompressed rat (Zhang et al., 2017b), a finding which supports a cause-and-effect relationship in which detached bubbles bring about endothelial injury.

### Microparticles (MPs)

Microparticles are present in the blood of any healthy individual. These MPs are tiny vesicles (0.1–1.0 μm) derived from pieces of cell membrane from leukocytes, erythrocytes, platelets, and endothelial cells. Exchange of markers between different MPs may cause a single MP to carry markers from various sources. Their number increases with stress, such as oxidative stress and apoptosis. After diving, there is an increase in circulating microparticles in the blood. Extensive research on this topic has been conducted in mice and divers (Thom et al., 2011, 2012, 2013). Some of the enlarged MPs contain gas (Yang et al., 2012; Thom et al., 2013). Thom et al. (2012) reported that there was a significant inverse correlation between post-diving bubble scores and MPs in divers. These MPs cause platelet aggregation and inflammation, and neutrophil activation. An association has been shown between MPs and decompression sickness (Thom et al., 2015). Madden and Laden (2009) even suggested that endothelial malfunction and MPs, not gas bubbles, may be the underlying cause of DCI. Enlarged MPs contain gas (Yang et al., 2012), are rich in iNOS, and contain NO_2_ (Thom et al., 2013). Eight percent of the micronuclei from normal mice contained gas, and Thom et al. (2013) suggested these may be the gas micronuclei from which bubbles develop after decompression. Pathological effects were related to these enlarged MPs. Bubbles caused the release of endothelial MPs from endothelial culture, and these specific MPs were associated with severe endothelial injury and pro-inflammatory cytokines (Yu et al., 2017). However, I find it difficult to accept that even with high iNOS activity, the short-lasting NO and the more water-soluble NO_2_, which easily crosses membranes and decomposes in water (Clean Air Technology Center, 1999; Signorelli et al., 2011), would create a pure gas phase from a solution of NO_2_. There was also no mention of the mechanism whereby the MPs are torn from the membrane during and after decompression. According to my understanding, it is more probable that NO and its oxidized derivatives would diffuse into an already existing gas phase. Therefore, I would suggest that pieces of membrane are carried along with detached bubbles, some of which completely lose their gas in the lung, whereas others retain some of the gas, enabling them to cross the lung capillaries. It is possible that DPPC settled preferably on iNOS-rich membrane to form the AHS, which made the enlarged MPs rich in iNOS. A possible explanation for the negative relationship between MPs and bubbles (Thom et al., 2012) may be that at some time after the dive, most of the bubbles lost their gas in the lung and continued to circulate as MPs, whereas the number of newly formed bubbles in the venous circulation had already been depleted. Thom et al. (2013) suggested that gas-containing MPs serve to initiate bubble growth, and that once formed, it is the bubbles that cause widespread physical damage. I would suggest the opposite, that nanobubbles at the AHS and detached bubbles create the decompression MPs. It may be that when a bubble formed at an AHS loses most of its gas in the lung and continues to circulate as an enlarged gas-containing MP, it will serve as a secondary gas micronucleus for further bubble growth in both arterial and venous blood.

### Decompression modeling

Completely comprehensive physiological decompression models should therefore take into account bubble formation at the AHS, both in the venous and distal arterial circulation, as well as the added risk of inflammation, blood clotting and neutrophil activation, due to venous bubbles which are transformed into MPs while passing through the lung, and secondary bubble expansion from these gas-containing MPs.

### Autoimmune diseases

A nanobubble gas phase in the path of the circulating blood may have far-reaching effects. Proteins with hydrophobic regions circulating in the blood will adhere to the gas phase-plasma interface. Deformation of their secondary and tertiary configuration will present them as foreign molecules or autoantigens. Normally hidden components of the intact protein (therefore unrecognized during thymus education), which are also present in a deformed protein, may be recognized as foreign as well, i.e., epitopes. This process has been proposed as a trigger for autoimmune diseases (Arieli, 2015). The presence of an autoimmune disease in homoeothermic, air-breathing vertebrates (DPPC can aggregate only in warm-blooded animals) will serve to increase autoimmunity, and the elevated risk of DCI with age may also be matched with the appearance of AHS. The same is true for the variable sensitivity to both diseases. If this hypothesis is proven correct, eliminating the AHS may provide protection against autoimmune diseases and DCI.

## Author contributions

The author confirms being the sole contributor of this work and approved it for publication.

### Conflict of interest statement

The author declares that the research was conducted in the absence of any commercial or financial relationships that could be construed as a potential conflict of interest.
